# High-flow nasal oxygen is the reference treatment in acute hypoxemic respiratory failure: Con

**DOI:** 10.1016/j.jointm.2024.12.005

**Published:** 2025-01-21

**Authors:** Gabriel Kemoun, Alexandre Demoule

**Affiliations:** 1 AP-HP, 26930, Groupe Hospitalier Universitaire APHP-Sorbonne Université, site Pitié-Salpêtrière, Service de Médecine Intensive et Réanimation (Département R3S), Paris F-75013, Île-de-France, France; 2 Sorbonne Université, INSERM, UMRS1158 Neurophysiologie Respiratoire Expérimentale et Clinique, Paris, France

**Keywords:** Acute respiratory failure, Intubation, Low-income country, High flow nasal cannula, Patient self-inflicted lung injury, Carbon emissions

## Abstract

Over the past decade and boosted by the coronavirus disease 2019 (COVID-19) pandemic, high-flow nasal oxygen (HFNO) has been increasingly used in the intensive care unit (ICU) to treat acute hypoxemic respiratory failure (AHRF). In this review, we show that despite this wide and rapid increase in the use of HFNO to treat AHRF, HFNO does not fulfill all the criteria of a “reference treatment”. First, there are some inconsistencies between the studies that provided a positive signal toward the possible benefit of HFNO in AHRF. The two high-quality studies were negative in terms of primary outcome although they provided promising signals in favor of HFNO in terms of secondary outcomes or unplanned secondary analysis. The significance of the only positive study suffers from notable limitations and other trials, conducted in COVID-19 and in immunocompromised patients, are definitely negative and do not even provide promising signals in favor of HFNO. Of note, authors of some of the large randomized controlled trials (RCTs) on HFNO have received grants or personal fees from manufacturers of HFNO devices. Second, meta-analyses do not show positive results regarding the efficacy of HFNO on mortality and recent guidelines do not support its use to improve this outcome, although they recommend HFNO use to reduce intubation rate. Third, HFNO is associated with risks that should be accounted for. There are concerns that HFNO may delay intubation, which is in turn associated with higher mortality and prolonged length of stay. In addition, with HFNO, high inspiratory effort may generate high lung strain and overstretch, a phenomenon termed patient self-inflicted lung injury (P-SILI). Fourth, there are concerns regarding access to HFNO in resource-limited settings. Fifth, there are also concerns regarding the deleterious environmental impact of HFNO due to the high volume of consumables and high oxygen flow, which remain to be precisely quantified and balanced with the potential reduction in intubation rate. Considering all these limitations, HFNO is not yet the reference treatment for AHRF.

## Introduction

Over the past decade, high-flow nasal oxygen (HFNO) has been increasingly used in the intensive care unit (ICU) to treat acute hypoxemic respiratory failure (AHRF). This rapid spread of HFNO to treat AHRF was boosted by the coronavirus disease 2019 (COVID-19) pandemic.

Despite this wide and rapid increase in the use of HFNO to treat AHRF, HFNO does not fulfill all the criteria of a “reference treatment”. The present review will discuss all the reasons why HFNO has not yet achieved the status of “reference treatment”. First, the results of the high-quality trials that have suggested a benefit of HFNO are conflicting. Hence, there remains a paucity of evidence to definitively support HFNO use in AHRF. As a consequence, recent guidelines do not strongly support the use of HFNO to reduce mortality in AHRF. Second, HFNO is associated with two major risks: to delay intubation and to cause patient self-inflicted lung injury (P-SILI). Third, questions arise regarding the global availability of HFNO. Finally, there are serious concerns about its environmental impact.

This review does not discuss the role and use of HFNO to treat acute or chronic respiratory failure and pulmonary edema, to prevent and treat post-extubation acute respiratory failure, and to secure intubation.

### Trials That Have Assessed the Potential Benefit of HFNO are Conflicting

Most large randomized controlled trials (RCTs) that have assessed the impact of HFNO on AHRF are of high quality. However, the results of these trials are conflicting. Although some of them have shown signals suggesting a possible benefit of HFNO on intubation or mortality, for statistical and methodological reasons, very few of them could conclude that HFNO was definitely beneficial. All major trials evaluating HFNO in AHRF are summarized in Table 1.

**Table 1:** Major RCTs evaluating HFNO in AHRF.

Study (year)	*n*	Population	Control	Primary outcomes	Secondary outcomes
Frat et al.^[^^1^^]^ (2015)	310	General P/*F* < 300 mmHg	COT; NIV	28-day intubation rate: HFNO 38%; COT 47%; NIV 50% (*P*=0.18)	90-day mortality: HFNO 12%; COT 23%; NIV 28% (*P*=0.02)* Intubation subgroup P/*F* < 200 mmHg: HFNO 35%; COT 53%; NIV 58% (*P*=0.009)*
Azoulay et al.^[^^2^^]^ (2018)	776	Immunosuppressed Oxygen flow ≥6 L/min	COT	28-day mortality: HFNO 36%; COT 36% (*P*=0.94)	28-day intubation rate: HFNO 39%; COT 44% (*P*=0.17) No benefit of HFNO on dyspnea and comfort
Ospina-Tascon et al.^[^^3^^]^(2021)	220	COVID-19 P/*F* < 200 mmHg	COT	28-day intubation rate: HFNO 34%; COT 51% (*P*=0.03)* 28-day clinical recovery: HFNO 78%; COT 71% (*P*=0.047)*	14-day mortality: HFNO 6%; COT 6% (*P*=0.90) 28-day mortality: HFNO 8%; COT 16% (*P*=0.11)
Grieco et al.^[^^4^^]^(2021)	109	COVID-19 P/*F* < 200 mmHg	Helmet-NIV	Days free of respiratory support: HFNO 18 days; helmet-NIV 20 days (*P*=0.26)	28-day intubation rate: HFNO 51%; helmet-NIV 30% (*P*=0.03)* Ventilator free days: HFNO 25; helmet-NIV 28 days (*P*=0.04)*
Bouadma et al.^[^^5^^]^(2022)	546	COVID-19 Oxygen flow ≥6 L/min	CPAP; COT	28-day intubation rate: HFNO 44%; COT 41%; CPAP 43% (*P*=0.85)	
Frat et al.^[^^6^^]^(2022)	711	COVID-19 P/*F* < 200 mmHg	COT	28-day mortality: HFNO 10%; COT 11% (*P*=0.60)	ICU mortality: HFNO 12%; COT 15% (*P*=0.25) 90-day mortality: HFNO 13%; COT 15% (*P*=0.56) Ventilator free days: HFNO 28; COT 23 days (*P*=0.07)
					28-day intubation rate: HFNO 45 %; COT 53 % (*P*=0.04)*
Perkins et al.^[^^7^^]^(2022)	1273	COVID-19 FiO_2_ >40%	CPAP; COT	30-day mortality and intubation rate: HFNO 44% *vs.* COT 45% (*P*=0.83) CPAP 36% *vs.* COT 44% (*P*=0.03)	

AHRF: Acute hypoxemic respiratory failure; COVID-19: Coronavirus disease 2019; COT: Conventional oxygen therapy; CPAP: Continuous positive airway pressure; HFNO: High-flow nasal oxygen; ICU: Intensive care unit; *n:* Number of patients; NIV: Non-invasive ventilation; P/F: Partial arterial oxygen pressure to inspired oxygen fraction ratio; RCTs: Randomized controlled trials.

⁎ Significant outcomes.

#### Comparison with conventional oxygen therapy (COT)

Multiple RCTs have compared HFNO to COT in various populations of AHRF patients, including the general population^[^^1^^]^ of course, but also immunocompromised patients ^[^[2], [8]^]^ and those admitted with AHRF due to COVID-19. ^[^[3], [5], [6], [7], [9]^]^ Of notice, there are more trials that have assessed the benefit of HFNO in subcategories of patients (immunocompromised and COVID-19) than in the general population, which is quite surprising, to say the least.

The first large RCT, the FLORALI trial,^[^^1^^]^ included patients with a partial arterial oxygen pressure to inspired oxygen fraction ratio (PaO_2_/FiO_2_) <300 mmHg. The primary outcome was the proportion of patients who required intubation 28 days after randomization. Patients were randomized into three groups: HFNO, non-invasive ventilation (NIV) with HFNO between NIV sessions or COT. The study included 310 patients. Regarding the primary outcome (intubation rate 28 days after randomization), the study was negative: 38% were intubated in the HFNO group, 47% in the COT group, and 50% in the NIV group (*P*=0.18). A positive outcome was only identified after the authors performed a *post hoc* unplanned exploratory sensitivity analysis in the subgroups of the 238 most severely hypoxemic patients, defined by PaO_2_/FiO_2_ <200 mmHg. Of notice, there were no PaO_2_/FiO_2_ strata at randomization. In these most severely hypoxemic patients, the authors found a significantly lower rate of intubation in the HFNO group (35%) than in the COT group (53%) or NIV group (58%) (*P*=0.009). Surprisingly, in the whole population (not only in the most severely hypoxemic patients), mortality 90 days after randomization, a secondary outcome among others, was significantly lower in the HFNO group (12%) than in the COT and the NIV group (23% and 28%, respectively, *P*=0.02). To summarize, the study was negative in terms of primary outcome (intubation rate), but positive in a *post hoc* analysis (without stratification) and positive in terms of a relevant secondary outcome. Subsequently, although this high-quality study provides interesting and promising positive signals in favor of the benefit of HFNO over COT and NIV, statistically and methodologically speaking, it cannot be considered positive.

The same investigators conducted a subsequent larger RCT in 711 COVID-19 patients, randomized to receive either HFNO or COT.^[^^6^^]^ Given the results of their previous trial, which suggested a benefit of HFNO on mortality rather than intubation and mostly in the most severely hypoxemic patients,^[^^1^^]^ they focused this second trial on patients with PaO_2_/FiO_2_ <200 mmHg and the primary outcome was mortality 28 days after randomization. The study was well-conducted, with good adherence to allocated treatments, well-balanced groups, and patients were actually severely hypoxemic with a median PaO_2_/FiO_2_ of 130 mmHg at inclusion. In terms of mortality, the primary outcome, the study was negative (10% in the HFNO group *vs.* 11% in the COT group, *P*=0.60). However, in contrast to their previous study,^[^^1^^]^ intubation rate by day 28, a secondary outcome, was significantly lower in the HFNO group than in the COT group (45% *vs.* 53%, *P*=0.04). This second large high-quality study was therefore also negative as there was no significant benefit of HFNO in terms of mortality, the primary outcome.

A third study, conducted in three ICUs in Colombia, showed similar results.^[^^3^^]^ Two hundred and twenty severely hypoxemic (PaO_2_/FiO_2_ <200 mmHg) COVID-19 patients were included and randomized to receive either HFNO or COT. The investigators used co-primary outcomes, intubation and time to clinical recovery within 28 days after randomization. This study was positive since it found a lower intubation rate and greater clinical recovery at day 28 with hazard ratios (HR) of 0.62 (95% confidence interval [CI]: 0.39 to 0.96) and 1.39 (95% CI: 1.00 to 1.92), respectively. Of notice, there was no significant difference in terms of mortality, a secondary outcome. However, although positive, this study suffers from important limitations. First, there was no adjustment for multiple comparisons. Second, 21 patients did not receive interventions and were not analyzed.

Other trials are definitively negative and did not even provide a positive signal suggesting a benefit of HFNO in AHRF. The large RECOVERY-RS trial conducted in the United Kingdom during the COVID-19 pandemic compared COT to HFNO and continuous positive airway pressure (CPAP).^[^^7^^]^ The primary outcome was a composite of intubation or mortality 30 days after randomization. Despite an early recruitment stop, 783 participants were included in the comparison of HFNO with COT. There was no significant difference in the primary outcome (44% in the HFNO group *vs.* 45% in the COT group, *P*=0.83). Of notice, mortality at 30 days was similar in the two groups (19% in the HFNO group *vs.* 20% in the COT group, *P*=0.66). However, this study suffers from significant limitations: early termination, unbalanced randomization, and crossover between groups. Another RCT conducted in France compared HFNO, CPAP, and COT in 333 COVID-19 patients.^[^^5^^]^ It evaluated simultaneously the benefit of dexamethasone with a 2×3 factorial plan. There was no significant difference for the cumulative incidence of intubation criteria at day 28 among the different groups (COT *vs.* CPAP: HR=1.08, 95 % CI: 0.71 to 1.63; COT *vs.* HFNO: HR=1.04, 95% CI: 0.69 to 1.55) or 60-day mortality (COT *vs.* CPAP: HR=0.97, 95% CI: 0.58 to 1.61, COT *vs.* HFNO: HR=0.89, 95% CI: 0.53 to 1.47).

Immunocompromised patients are a population of the utmost interest since AHRF is frequent and intubation is associated with a major risk of mortality. A large trial has compared HFNO to COT,^[^^2^^]^ which included 776 patients. The primary outcome was mortality within 28 days after randomization. The trial did not show any difference between the two groups in terms of mortality (HR=0.98, 95% CI: 0.77 to 1.24) and even intubation rate (39% in the HFNO group *vs.* 44% in the COT group, *P*=0.17).

**Figure 1:**
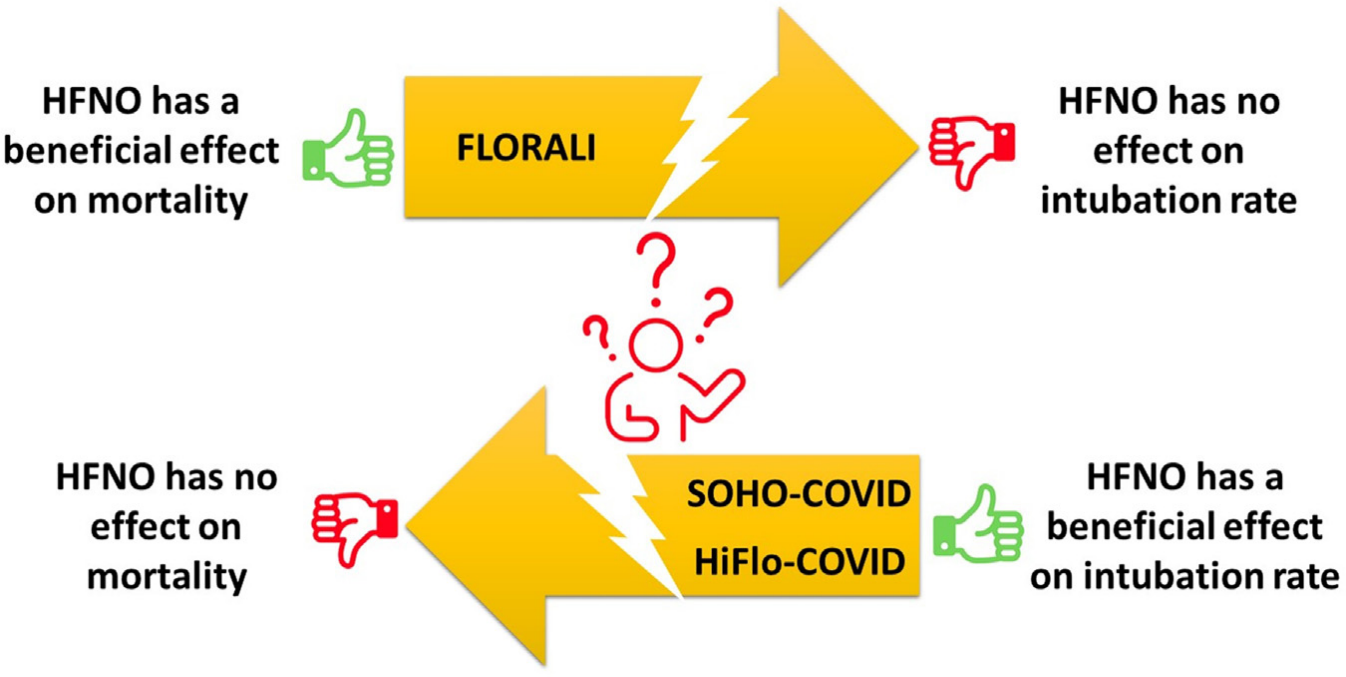
Major randomized control trials show inconsistent results. The first large study evaluating HFNO in AHRF (the FLORALI study^[^^1^^]^), although it failed to demonstrate a significant effect on intubation rate, did find a reduced mortality rate. Conversely, the two subsequent studies evaluating the same question (the SOHO-COVID^[^^6^^]^ and HiFlo-COVID^[^^3^^]^ studies) surprisingly demonstrated the exact opposite: a reduction of the intubation rate, but no reduction of mortality. AHRF: Acute hypoxemic respiratory failure; HFNO: High-flow nasal oxygen.HiFlo-COVID: Effect of High-Flow Oxygen Therapy vs Conventional Oxygen Therapy on Invasive Mechanical Ventilation and Clinical Recovery in Patients With Severe COVID-19: A Randomized Clinical Trial; SOHO-COVID: Standard Oxygen Versus High Flow Cannula Oxygen Therapy in Patients With Acute Hypoxemic Respiratory Failure.

To summarize, there are some inconsistencies between the studies that provided a positive signal toward the possible benefit of HFNO in AHRF. The two high-quality studies were negative in terms of primary outcome. In terms of secondary outcome, one of them showed a benefit on mortality but not on intubation^[^^1^^]^ while the other showed the contrary, a benefit on intubation but not on mortality^[^^6^^]^ (Figure 1). The only positive study, however, suffered from notable limitations.^[^^3^^]^ Other trials, in COVID-19 and in immunocompromised patients,^[^^2^^,^^5^^,^^7^^]^ are definitely negative and did not even provide a positive signal suggesting a benefit of HFNO. Finally, it is worth mentioning that authors of some of the large RCTs on HFNO have received grants or personal fees from manufacturers of HFNO devices.^[^^10^^]^

#### Comparison with CPAP or NIV

Although the FLORALI trial showed no difference between HFNO and NIV in terms of intubation rate and a significant reduction in mortality rate in the HFNO group as compared to the NIV group, other studies showed opposite results.

A *post hoc* analysis of the RECOVERY RS trial compared CPAP to HFNO.^[^^7^^]^ This comparison showed that, as compared to HFNO, CPAP reduced intubation or 30-day mortality, the primary outcome, with an absolute difference of −10% (95% CI: −18% to −2%). However, the aforementioned limitations of the original trial must be taken into account.

The HENIVOT trial compared helmet-NIV to HFNO in 109 severely hypoxemic (median PaO_2_/FiO_2_ 102 mmHg) patients.^[^^4^^]^ The primary outcome was the number of days free of respiratory support. There was no significant difference in the primary outcome (20 days for helmet-NIV *vs.* 18 days for HFNO, *P*=0.26). However, the intubation rate within 28 days was significantly lower in the helmet-NIV group than in the HFNO group (30% *vs.* 51%, *P*=0.03).

These two studies suggest that CPAP and helmet-NIV might be superior to HFNO in AHRF. Not only the benefit of HFNO over COT in AHRF is not clearly demonstrated, but also non-invasive respiratory support like helmet-NIV and CPAP might be superior to HFNO, at least to reduce intubation rate.

### Meta-Analyses Show Mixed Results

These findings have been computed in meta-analyses that combined data from ICU and emergency department patients, encompassing immunosuppressed and non-immunosuppressed individuals, and even included studies using a lower flow rate of 35 L/min (while most studies and clinical practice employ higher flows up to 70 L/min). A first meta-analysis reported no significant differences in mortality rate, length of stay, or even comfort when comparing HFNO with COT.^[^^11^^]^ However, there was a positive impact of HFNO on the intubation rate (risk ratio 0.74–0.99), with moderate certainty of evidence. The meta-analysis performed in the more recent European Respiratory Society guidelines showed similar findings, with no significant effect of HFNO on mortality compared to COT (moderate certainty of evidence) and no significant differences when compared to NIV (risk ratio=0.89, 95% CI: 0.77 to 1.02).^[^^12^^]^ Of notice, the intubation rate did not differ significantly between HFNO, COT and NIV.

Recently, the European Society of Intensive Care Medicine (ESICM) produced guidelines on the management of acute respiratory distress syndrome.^[^^13^^]^ Regarding HFNO, these guidelines reached the ambiguous conclusion that HFNO was recommended over COT to prevent intubation (with a moderate level of evidence) but that experts were unable to make a recommendation for or against the use of HFNO over COT to reduce mortality (with a high level of evidence). These conclusions were based on a meta-analysis showing that, compared to COT, there was no statistical difference in mortality for HFNO (relative risk=0.95, 95% CI: 0.82 to 1.09) but a beneficial effect in preventing intubation (relative risk=0.89, 95% CI: 0.81 to 0.97). A subsequent meta-analysis showed similar findings.^[^^14^^]^ Although preventing intubation is an important goal of care, the ultimate goal of non-invasive respiratory support like HFNO is to reduce mortality. In addition, it is difficult to understand how a reduction in the intubation rate does not translate into a reduction in the mortality rate.

### Could HFNO be Deleterious?

To explain why the reduction in intubation rate does not translate into a reduction in mortality, it has been suggested that, in some cases, HFNO may be deleterious and hence worsen the outcome. This deleterious impact of HFNO could involve two mechanisms: first, delayed intubation and, second, P-SILI.

#### High flow oxygen might delay intubation

To explain why the reduction in intubation rate does not translate into a reduction in mortality, it has been proposed that there is a potential detrimental effect of delayed intubation in patients receiving HFNO.

In 2015, a pioneer study assessed the outcome of patients intubated after HFNO failure, accounting for time to intubation. One hundred and thirty patients intubated within 48 h of HFNO were compared to 45 patients intubated >48 h after HFNO initiation.^[^^15^^]^ Propensity-adjusted analysis revealed an association between late intubation and increased ICU mortality (67% in the late intubation group *vs.* 39% in the early intubation group, *P*=0.001). However, it should be noted that the median time to intubation was 126 h in the late intubation group *vs.* 10 h in the early intubation group, which suggests that the two populations were different.

Several other studies support an association between delayed intubation and poor outcomes. While most of them have included mixed populations of patients receiving HFNO or NIV,^[^[16], [17], [18]^]^ others have focused specifically on patients receiving HFNO only.^[^^19^^,^^20^^]^ In a retrospective cohort study of 2720 COVID-19-infected patients,^[^^20^^]^ mortality steadily increased as a function of time to intubation. Hospital mortality was higher in patients intubated >24 h after the initiation of HFNO (67% *vs.* 49%, *P* < 0.0001). These results, which suggest a harmful effect of late intubation, need to be confirmed through a comparative study designed to assess the specific role of HFNO in delaying intubation.

Although the association between late intubation and increased mortality may be explained by the higher severity of patients who received late intubation, another explanation is that prolonged exposure to HFNO may cause lung injury. The mechanism through which spontaneous breathing may cause lung injury in patients with severe AHRF is called P-SILI.

#### High flow oxygen might expose patients to self-inflicted lung injury

P-SILI is an emerging concept, which is supported by respiratory physiology and experimental studies.^[^^21^^,^^22^^]^ Briefly, as a consequence of the initial lung injury (pneumonia, etc.), the respiratory drive increases, which in turn generates high, if not very high inspiratory efforts. These high inspiratory efforts increase transpulmonary pressure, leading to greater lung stress, which in turn promotes lung injury in a manner similar to ventilator-induced lung injury (VILI). These high inspiratory efforts may also cause phenomena like pendelluft, lung overstretch, atelectrauma, and increased vascular transmural pressure. Preliminary data have suggested that P-SILI could be involved in NIV failure. Indeed, retrospective and prospective studies have shown that, in patients receiving NIV for AHRF, high tidal volume (>9 mL/kg or 9.5 mL/kg) was independently associated with a higher risk of intubation.^[^[23], [24], [25]^]^ Because the routine monitoring of tidal volumes is not possible under HFNO, the association between high tidal volume and intubation could not be studied in patients receiving HFNO.

A crossover physiological study that compared HFNO to helmet-NIV and helmet-CPAP in patients with AHRF suggests however that HFNO may cause P-SILI.^[^^26^^]^ In this study, helmet-NIV resulted in a decrease in esophageal pressure, but transpulmonary pressure was not different across conditions. However, pendelluft and end-expiratory lung impedance (measured by electrical impedance tomography) was higher with HFNO than with helmet-CPAP and helmet-NIV. In addition, HFNO resulted in higher dynamic lung strain and lung overstretch than helmet-CPAP. This physiological data suggests that HFNO may be associated with more pendelluft, lung strain, and lung overstretch, which are potentially involved in P-SILI.

Finally, the prolonged exposure to a non-invasive respiratory support technique that causes P-SILI may explain why patients intubated after a longer duration of HFNO exhibit after intubation more altered respiratory system mechanics and gas exchange. Indeed, in a study that included 75 COVID-19 patients intubated after HFNO failure, patients intubated after 1.27 days had higher driving pressure, lower static compliance, and higher ventilatory ratios (though these results were not statistically significant) than patients intubated before 1.27 days.^[^^27^^]^

To summarize, delayed intubation and prolonged exposure to high lung strain and overstretch may explain how HFNO may cause P-SILI and hence be deleterious.

### HFNO is not Easily and Largely Available in Low and Lower Middle Income Countries

The accessibility of HFNO in lower income countries is an under-discussed topic. The initial cost of purchasing devices, consumables, and ongoing maintenance presents a significant hurdle, especially for healthcare systems already struggling with access to mechanical ventilation (both invasive and non-invasive).^[^^28^^]^ Furthermore, HFNO requires a high oxygen flow rate compared to COT, which necessitates a robust oxygen delivery infrastructure. In low and lower middle income countries, access to oxygen itself is often limited, with up to a quarter of hospitals lacking supplemental oxygen availability.^[^^29^^]^ This is a major concern,^[^^30^^,^^31^^]^ as highlighted by the recent COVID-19 pandemic.^[^^32^^]^ Given the uncertain benefit of HFNO compared to COT in AHRF, its use seems difficult to advocate for in resource-limited settings. Of notice, assessing the cost/effectiveness of HFNO needs to consider that it may reduce the intubation rate.

Two ongoing clinical trials are currently investigating HFNO against standard flow oxygen therapy in those settings, one in Uganda (Adult Respiratory Failure Intervention Study Africa ARISE-AFRICA, clinicaltrials.gov ID: NCT04693403) and one in Rwanda, Kenya and Malawi (Building Respiratory Support in East Africa Through High Flow Versus Standard Flow Oxygen Evaluation [BREATHE], clinicaltrials.gov ID: NCT05754034).

### Environmental Impact of HFNO

The growing concern over climate change necessitates a critical evaluation of the environmental impact of healthcare practices. Global warming has direct and indirect impacts on human health, which in turn affects intensive care.^[^^33^^]^ Notably, ICUs generate a significantly higher carbon footprint compared to general wards,^[^^34^^]^ up to 178 kg of carbon dioxide equivalent (CO_2_-e) emissions.^[^^35^^]^ Estimates suggest that the CO_2_ emissions footprint of a single ICU patient is equivalent to that of a four-person household^[^^36^^]^ and that the daily waste generated by one patient can be up to 13.7 kg/day.^[^^37^^]^

Consumables and oxygen delivery contribute significantly to this burden. Initiatives to reduce the carbon footprint of ICUs include the reduction and optimization of consumable use, especially single-use materials to reduce waste.^[^^38^^]^ The CO_2_ emissions related to HFNO devices have not yet been calculated, but it is likely that it is higher than the CO_2_ emissions of COT. Another initiative advocated is minimizing unnecessary oxygen therapy by exploring lower flow options.^[^^39^^]^ A recent life cycle assessment study in the United States evaluated the burden of medical oxygen use.^[^^40^^]^ They estimated that one patient consuming oxygen at a flow rate of 2 L/min produced between 0.49 (in Ontario, Canada) and 5.7 kg CO_2_-e (in China) per day depending on the location of the production site and electricity grid. Given that HFNO requires high flow rates, up to 70 L/min, it should be associated with high CO_2_-e emission.

This environmental impact of HFNO needs to be more precisely evaluated, considering both the effects of consumables and the implications of high oxygen flow balanced with the potential impact on intubation rate.

## Conclusions

**Figure 2:**
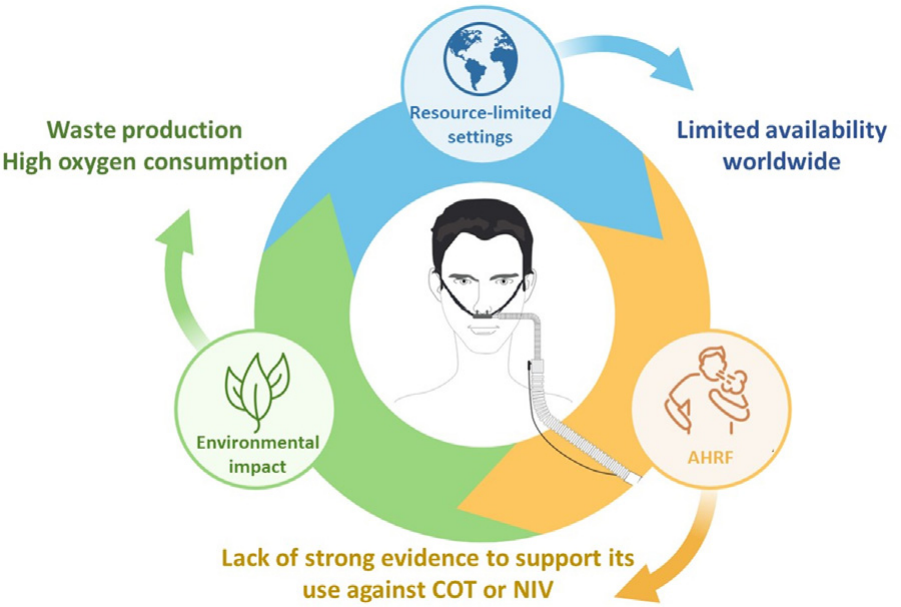
Limitations remain to consider HFNO as a reference treatment. There are several limitations to consider before adopting HFNO as the reference treatment for AHRF. First, a review of the literature reveals a lack of strong evidence supporting its benefits compared to COT or NIV. Second, HFNO availability remains limited in resource-constrained settings. Third, carbon dioxide emissions associated with HFNO use may be higher than emissions associated with COT. AHRF: Acute hypoxemic respiratory failure; COT: Conventional oxygen therapy; NIV: Non-invasive ventilation.

Despite its widespread adoption in the ICU and numerous RCTs, there is no strong evidence to support that HFNO is beneficial in AHRF compared to COT, NIV, or CPAP. Recent guidelines recommend the use of HFNO to reduce intubation rate, but not to reduce mortality, which is quite surprising in terms of clinical practice. An ongoing large clinical trial comparing HFNO to COT may help to solve these issues (Standard Oxygen Versus High Flow Nasal Cannula Oxygen Therapy in Patients with Acute Hypoxemic Respiratory Failure [SOHO], NCT04468126). However, concerns will remain regarding access to HFNO in resource-limited settings and its deleterious environmental impact. Considering all these limitations, HFNO is not yet the reference treatment for AHRF. A graphic summary of these limitations is displayed in Figure 2.

## CRediT authorship contribution statement

**Gabriel Kemoun:** Writing – review & editing, Writing – original draft, Methodology, Formal analysis, Conceptualization. **Alexandre Demoule:** Writing – review & editing, Writing – original draft, Supervision, Methodology, Investigation, Conceptualization.
